# Classification of skin phenotypes caused by diabetes mellitus using complex scattering parameters in the millimeter-wave frequency range

**DOI:** 10.1038/s41598-017-06034-0

**Published:** 2017-07-19

**Authors:** Fabian Dornuf, Pedro Martín-Mateos, Blanca Duarte, Bernhard Hils, Oscar Elias Bonilla-Manrique, Fernando Larcher, Pablo Acedo, Viktor Krozer

**Affiliations:** 1 Physics Institute, Goethe University Frankfurt am Main, 60438 Frankfurt am Main, Germany; 2 Department of Electronics Technology, Universidad Carlos III de Madrid, Leganes, Madrid, 28911 Spain; 3 Epithelial Biomedicine Division, CIEMAT, Avenida Complutense 40, Madrid, 28040 Spain; 4 Centro de Investigación Biomédica en Red de Enfermedades Raras (CIBERER), Madrid, Spain; 5 Department of Bioengineering, Universidad Carlos III de Madrid, Leganes, Madrid, 28911 Spain; 6 Instituto de Investigaciones sanitarias de la Fundación Jimenez Diaz (IIS-FJD), Madrid, Spain

## Abstract

The pathological skin phenotype caused by hyperglycemia is an important indicator for the progress of diabetes mellitus. An early detection of diabetes assures an early intervention to regulate the carbohydrate metabolism. In this publication a non-invasive detection principle based on the measurement of complex scattering parameters in the millimeter-wave frequency range is presented. The measurement principle provides evidence of the applicability for the identification of different glycemic states in animal models. The method proposed here can be used to predict diabetes status in animal models and is interesting for application on humans in view of safeness of millimeter-wave radiation. Furthermore the complex scattering parameters give important information about the anatomic varieties between the analyzed skin samples of the different mice strains. In contrast to other methods, our approach is less sensitive to skin variations between animals.

## Introduction

In modern medicine diabetes mellitus is one of the most researched diseases because of its wide spread in society. The sustained hyperglycemic state, which is caused by the inadequate regulation of the blood glucose level, leads to several pathologic changes like cardiovascular diseases or nephropathy^1–3^. Although, the current blood glucose is the main parameter to evaluate the acute diabetic state^4^, several other indicators, like glycated hemoglobin (HbA1c) have proved to be reliable markers for the long time evaluation of the disease^5^. The probability for cellular malfunctions caused by sustained hyperglycemia increases without proper glycemic control. The formation of advanced glycation end-products (AGEs) is one of the important effects caused by the sustained hyperglycemia^6–10^, considering that high glucose levels lead not only to covalent adducts with plasma proteins but also with extracellular matrix proteins such as collagen^9–11^. Apart from the serial monitoring of HbA1c only few other markers have been described for glycemic control like glycated albumin or albumin-corrected fructosamine^12–15^. Early detection of diabetes is an important aspect to counteract the disease in an early and plannable state and may therefore lead to improved patient recovery and lessen negative impact on the health care system.

In addition, *in-vivo* non-invasive methods of early diabetes detection are preferred in real life settings. Among those methods, millimeter-wave and THz methods have received recent attention due to minimum biological interaction with the host and hence safe operation of low-power millimeter-waves and THz radiation, see for example^16, 17^.

In a previous publication^17^ we have demonstrated that an *in-vivo* non-invasive measurement principle based on mm-wave spectroscopy through a skin fold of mice can distinguish between mice strains of different hyperglycemic states using statistical methods. We showed that a principal component analysis (PCA) algorithm can identify mice stems with different hyperglycemic states exploiting the amplitude of the transmission measurement through the skin. During the same measurements not only the transmission amplitudes of the mm-wave signal was recorded, but also the complex information of amplitude and phase for the transmission through the skin and the reflection at the skin surface.

In the present publication we want to demonstrate that a combination of the amplitude and phase information for the transmission and reflection measurements can be used to identify the different metabolic animal features using a deterministic discrimination approach. In contrast to principal component analysis (PCA) or partial least squares regression (PLS), which uses only the amplitude information of the transmission measurement^17^, we suggest here to utilize the amplitude and phase of the reflection and transmission measurements. In the last publication we showed a mathematical model using uncalibrated data. We demonstrate here, that using amplitude and phase information of the reflected and transmitted calibrated signals, respectively, provides sufficient information for the early detection of diabetes mellitus providing deterministic discrimination of the animal hyperglycemic states. We employ complex scattering parameters, which represent the amplitude and the phase of the reflected and transmitted signals relative to the incoming signal. These parameters are advantageous in terms of online monitoring and ease of data processing. The complex scattering parameters do not only provide the opportunity to identify and differentiate the glycemic states without statistical methods, the calibrated data provide also additional information about the thickness and the tissue properties of the measured skin samples and can be used to evaluate the skin structures responsible for the different responses for several strains. This is achieved at the expense of necessity of a calibration procedure, which however is a standard task in all measurements employing millimeter-wave S-parameters.

## Results

The mice strains, which were used for these measurements, can be subdivided into three different groups: control, diabetic and obese groups (see methods section). The control mice, which were present as nude mice, white-haired, and black-haired, exhibit a normal skin phenotype due to absence of sustained hyperglycemic states. The diabetic mice showed differences in the skin due to a high glucose level over a long period of time. The obese mice, were expected to also have a stressed skin phenotype caused by a higher blood glucose level compared to the control animals. Our goal has been to show that it is possible to separate all three categories based on deterministic discrimination of the amplitude and phase information, respectively. We demonstrate that it is also possible to distinguish between the mild diabetic obese animals and the strongly diabetic animals, which leads us to the conclusion that these mice strains have different manifestations in the skin phenotype and as a consequence varying reflection and transmission.

We will show below that the differences in reflection and transmission of the signals through the skin fold are indeed a consequence of the different manifestations of the glycemic states. To exclude that the present skin phenotype is caused only by the genetic predisposition and not by the different glycemic states, two groups of animals were used to modify the glycemic state in contrast to the genetic predisposition. Therefore, three of the nude control mice were chemically diabetized to develop a hyperglycemic state as the genetic diabetic mice. Also, two of the leptin deficient obese mice were treated with a leptin pump to restore a normoglycemic state and body weight similar to control mice.

In this discussion the complex scattering parameters for the transmission through a skin fold, S(2, 1), and the reflection at the skin interface, S(1, 1), are referred to amplitude and phase signals of two simple calibration standards. We demonstrate that a full two-port calibration procedure is not required for the measurements shown below and a simplified version of the calibration procedure is sufficient to uniquely discriminate between the three animal categories using amplitude and the phase data only.

**Figure 1:**
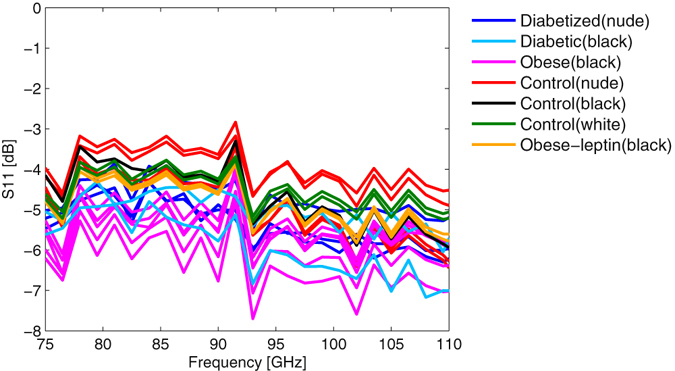
Amplitude of the reflection coefficient S(1, 1) versus frequency for the different mice strains.

First, we want to concentrate on the results of the complex reflection coefficient S(1, 1). As can be seen in Fig. 1, the amplitude of the reflection coefficient shows a small separation between the different glycemic states of the mice. The diabetic and the obese mice show a slightly lower reflection (more negative in dB scale) than the control animals. Interestingly, healthy animals, independent of the skin features (black, hairy, etc.), exhibit very similar reflection coefficients of the order of −3 to −4 dB. The difference in the reflection coefficient data to diabetized animals is up to 3 dB, which means a factor of two in linear scale. Further, it can also be observed that the reflection coefficient decreases with increasing frequency, which reflects the decrease in the permittivity of the skin with increasing frequencies. The spectral variation of the amplitude values of the reflection coefficient are due to the incomplete calibration of the full setup. It is important to mention that an extraction of the spectral properties of the reflection measurement would require a full calibration procedure of the measurement system.

**Figure 2:**
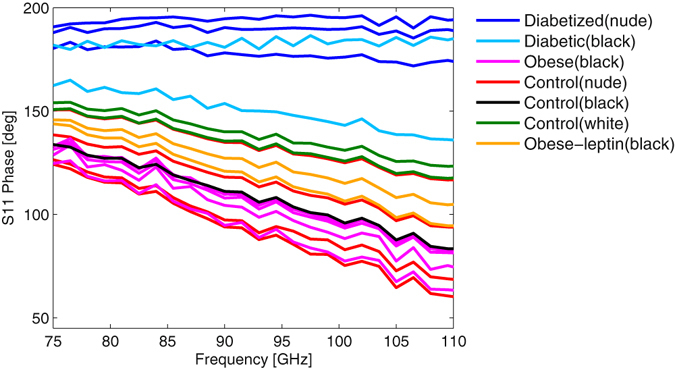
Phase of the reflection coefficient S(1, 1) versus frequency for the different mice strains.

If we also consider the phase of the reflection coefficient signal in Fig. 2 the separation in this component is more significant. The reflection coefficient is referred to the reflection of a polished metal plate which exhibits a phase of 180°. The diabetic mice show a similar phase near 180°, nearly independent of frequency, which indicates that diabetic mice epidermal phenotype is similar to a highly conductive medium exhibiting some losses. This has an implication on the choice of the model presented below. In contrast, the control mice indicate a phase shift below 180° against the reflection standard. Several important conclusions can be drawn from the results above. The diabetic and healthy animals can be clearly distinguished from the data in a concise manner without statistical methods. This distinction can be considered generic and can be applied in a blind test when the glycemic state of the animal is not known. The amplitude of the reflection coefficient will always be higher, in the order of −3 dB to −4 dB for a healthy animal with the according phase ranging between 100°–150°, while diabetic animals will exhibit a reflection coefficient 1–3 dB below the healthy animals or around −5 dB to −7 dB in absolute values and a phase of around 180° independent of frequency.

**Figure 3:**
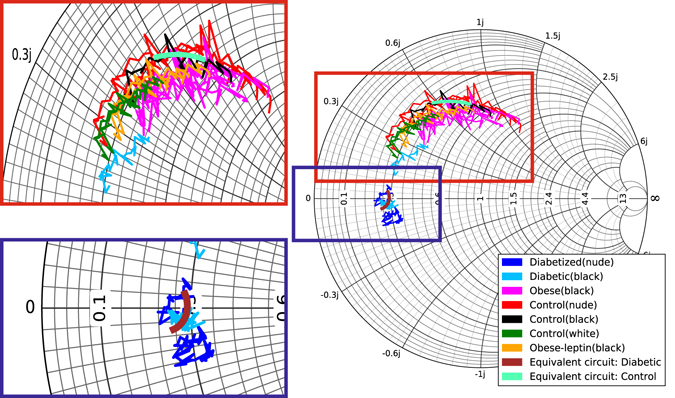
Measured reflection coefficient S(1, 1) in the complex plane as a function of frequency for control, diabetic, and obese mice together with the reflection coefficient of the equivalent circuit for diabetic and control animals (Fig. 4).

**Figure 4:**
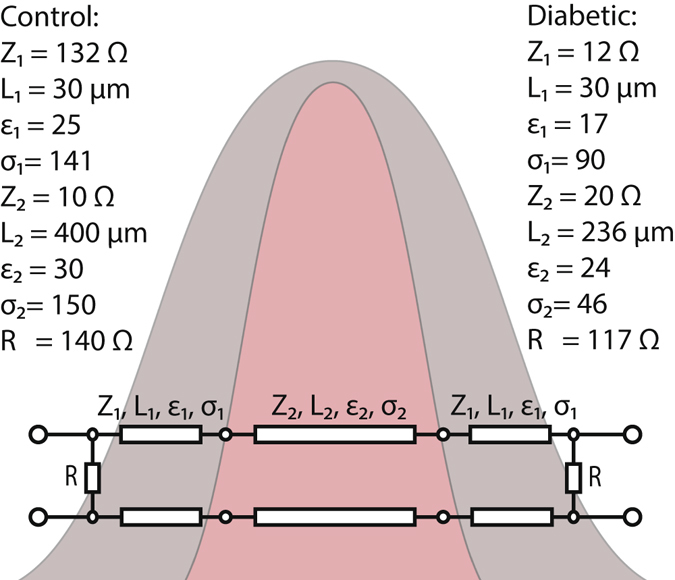
The proposed transmission line model.

For diabetic and healthy mice the separation is clearly directly visible in both amplitude and phase. The classification of the obese is only possible with a combination of the two parameters. The obese animals exhibit a reflection coefficient amplitude close to that of diabetic mice, while showing a phase behavior resembling that of healthy mice. This different behavior in amplitude and phase for the obese animals allows a separation to the diabetic and healthy mice and indicates a different skin phenotype for this group. It should be emphasized that in contrast to infrared (IR) techniques, millimeter-waves penetrate the skin to a considerable depth and hence several layers contribute to the overall reflected signal. Which layer is decisive for the response can be determined from the analysis of the reflection coefficient in the complex plane (Smith chart). In Figs 3 and 4 we assume a transmission line model explaining the reflection process for the different mice strains exemplary for a control and a diabetic mouse.

**Figure 5:**
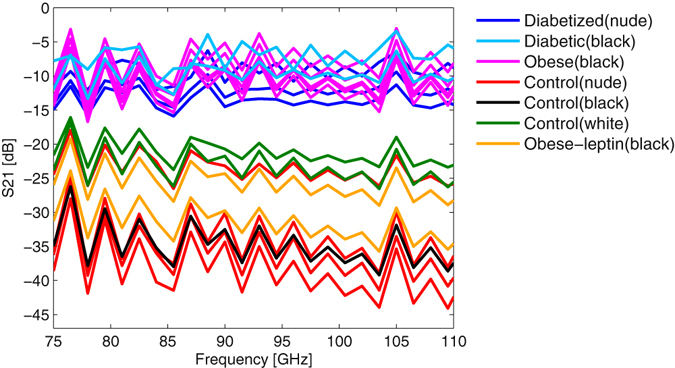
Amplitude of the transmission coefficient S(2, 1) versus frequency for the different mice strains.

The equivalent circuit is based on a simple model containing two components (epidermis and dermis), which are represented by transmission lines with an impedance Z, a length L, a dielectric constant ε and a dielectric loss caused by conductivity σ. Obviously, the transmission line impedance and dielectric constant are interrelated to each other, however the dielectric constant and conductivity represent the skin phenotype rather than direct water content. To consider the losses produced at the skin surface a shunt resistance R has also been inserted into the equivalent circuit. The model for the control animal shows a relatively high impedance for the first skin layer of 132 Ω but a small impedance for the second layer of 10 Ω, while the dielectric constant remains nearly unchanged. Furthermore, these animals show high dielectric constants and high conductivity losses in both skin layers. If we consider that the wave, which reaches the edge of the waveguide is both propagated through the skin fold into the opposite waveguide and is radiated in all direction, this behavior of the equivalent circuit can be explained as follows. The high impedance of the transmission line representing the outer skin for healthy animals and the high conductivity losses indicate that the wave is strongly attenuated in the skin. The wave propagates from the waveguide into the skin layer and is then radiated according to standard radiation law at the end of an open-ended waveguide. Therefore, the impedance of that line is slightly lower as compared to the wave impedance in the waveguide, which is in the order of *Z*_W_ ≈ 200 Ω. The low impedance of the second skin layer in the circuit behaves nearly as a short-circuit so that most of the wave is reflected back and only a small amount of wave is transmitted through the skin fold (compare Fig. 5).

The diabetic mice show different wave propagation behavior. The impedance of the first skin layer for diabetic mice is decreased to a value of 12 Ω and also the losses of the conductivity decrease drastically. This indicates that the attenuation process in the skin of the diabetic mice is weaker than in the healthy animals. Furthermore the increase in the impedance of the second skin layer from 10 Ω to 20 Ω indicates that the diffuse radiation in the second skin layer is more distinct in these skin samples compared to the healthy animals.

In our previous paper^17^, we estimated the thicknesses of the skin of control and diabetic mice to ~400 µm and ~300 µm, respectively, which agrees fairly well with the simple model presented here. Possible discrepancies could be the simplicity of the described model, which would require a more advanced modelling of the wave propagation inside the skin layers. On the other hand, the analysis in the complex impedance plane (Smith chart) presented here, unequivocally determines the model structure presented in Fig. 4, as neither a shunt nor series impedances and admittances can explain the measured data.

If we compare the measurements of the amplitude of the transmission (Fig. 5) with that of the reflection amplitude, the transmission amplitude shows a significant separation between the different mice strains. The diabetic and the obese mice have a stronger transmission (lower attenuation) than the control mice. In fact, the transmission in healthy mice is at least 10 dB lower as compared to genetically diabetic and diabetized mice and is typically in the order of −30 dB with little variation across the frequency range considered here. This is in full accordance with the reflection measurements presented above.

**Figure 6:**
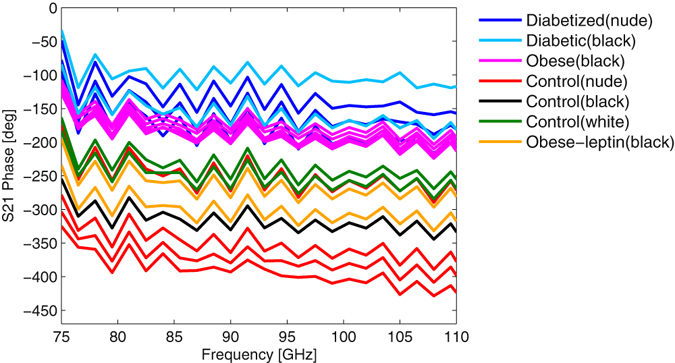
Phase of the transmission coefficient S(2, 1) versus frequency for the different mice strains.

As previously described^17^, the thickness of the skin fold of the obese mice is nearly double the thickness of the diabetic animal skin, but show similar behavior in the amplitude of the transmission, which indicates a difference in the composition of the skin and the respective layers. Therefore, we hypothesize that obese animals exhibit changes in the dermis or adipose layers of the skin^16, 18–20^. The phase of the transmission coefficient (Fig. 6) also confirms this observation. The phase shift in transmission measurements is referred to the situation, in which the two waveguides touch each other without the skin fold (thru standard). The obese and diabetic animals show only a small phase shift with respect to this thru standard. The control mice have a higher phase shift compared to the optimal transmission situation with the thru standard. In fact is evident that control animals exhibit a phase shift of about 360°, which corresponds to a layer thickness of 300 µm at 100 GHz and assuming a permittivity of ε_r_ = 25. This agrees well with the skin thicknesses presented above and determined by us in our previous publication^17^. These measurements also confirm that the parameters where properly chosen in the equivalent circuit model. We would like to emphasize that the equivalent circuit is a physical model and its parameters are based on physical properties of wave propagation through the skin fold.

## Discussion

We want to provide a rationale which anatomical changes in the skin of the mice lead to the measured scattering parameters illustrated above. First, we describe the categorization procedure for the different mice strains. On one hand, the control mice of the different strains were all grouped together and also the obese mice with the leptin pump show similar signals as the control mice. The mice, which were chemically diabetized classify into the diabetic group and not into the control group.

There is a clear evidence that the anatomical condition of the skin is not caused by the genetic predisposition but the different glycemic states. The results of the reflection coefficient measurements give some evidences about the composition of the skin layers. In Figs 3 and 4 we provide a simple physical skin model consisting of two different layers, the epidermis and the dermis. The equivalent circuit indicates that the skin of the control animals has a high attenuation which leads to a small transmission through the skin fold. In the skin of the diabetic animals the conductivity and also the dielectric constants for both skin layers are smaller. This indicates dry skin phenotypes and also a less dense composition of the skin. Also, a thinner skin thickness is depicted from this equivalent circuit, which is supported by thickness measurements mentioned in ref. 17. For the obese mice the smaller reflection amplitude might originate from dryness of the epidermis or a higher amount of sub-epidermal fat, which may also lead to differences in the reflection in contrast to the control mice.

The measured transmitted wave propagates through deeper skin layers as compared to reflection measurements. The transmitted signal has to propagate from the surface of a skin layer through the dermis and adipose volume, the epidermis and the outer skin again. The diabetic and the obese mice show a higher transmission amplitude and also a smaller phase shift compared to the healthy mice. This indicates a lower amount of fluid in the skin samples of these animals, as this is the major contribution to attenuation at millimeter-wave frequencies^17^, which is also observed in the higher conductivity loss in the control animals. Due to the higher phase shift in the transmitted signal, the healthy mice should show a higher dielectric constant in the dermis, which is indicated by the parameters of the equivalent circuit. In former studies^20–22^ it was shown that the dermis of obese mice have a smaller density of collagen structures which leads to decreased elasticity and an increased aging process of the skin. In contrast to healthy mice a less dense collagen net can potentially lead to differences in the dielectric constant and consequently to a higher transmission.

**Figure 7:**
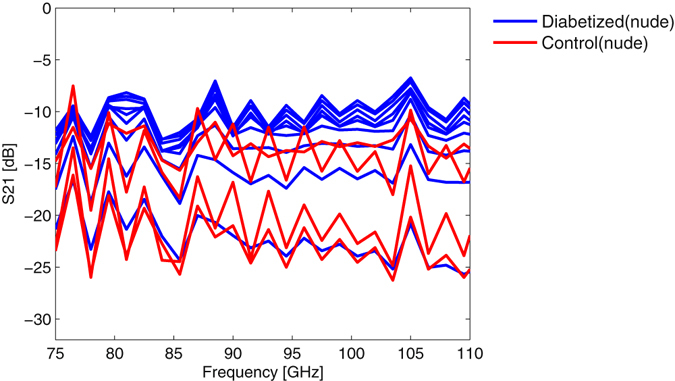
Amplitude of the transmission coefficient S(2, 1) versus frequency for diabetized and control mice, one week before the data in Fig. 5.

An interesting aspect is the variation of the mice strains over time. All the data shown above have been measured on one day to have better comparability. As we mentioned before, some of the nude control mice were treated chemically to develop diabetes. In addition to the measurements shown above, these mice were subject to repetitive measurements on several consecutive days to control the diabetization process. Figure 7 shows the amplitude of the transmission one week before the acquisition of the data in Figure 5. It can be seen that the two groups of mice show a better separation one week after the first measurement. This might indicate that published results on the instantaneous glucose level or glycemic state might be misleading as the changes in tissue properties require some time evolution to manifest themselves in non-invasive millimeter-wave measurements. The fact that diabetized mice have a similar skin phenotype after 12 days compared to genetic diabetic mice with an age of 6 months indicates that proper consideration of the age and also the speed of the metabolism is crucial to reliable explanation in further studies.

In our last publication we presented a robust measurement principle, which is capable of distinguishing between different glycemic states in animal models, using the transmission amplitude only in the mm-wave frequency range and PCA analysis.

The use of complex scattering parameters in the present study provides not only the opportunity to differentiate between the analyzed mice strains without statistical methods such as PCA, but also enables the analysis of the skin composition from the amplitude and phase of reflection and transmission signals. This is exciting as now we can offer two approaches for the non-invasive *in-vivo* determination of glycemic states with either a simple amplitude measurement with mathematical post-processing of the data using statistical analysis or with amplitude and phase measurements with real-time readout without the necessity for statistical analysis and with deterministic classification capabilities.

The more complex amplitude and phase measurements require accurate calibration of the instrumentation at millimeter-wave frequencies. In future work, the hypothesis of the skin constitution has to be proven by further experiments. Also alternative techniques like histopathological studies have to be done to evaluate the actual changes in the skin under sustained hyperglycemic states. It is also required to determine the metabolic alterations responsible for the drastic changes in the propagation properties in diabetic, obese and control animals.

## Methods

### Strains of the analyzed animal model

**Figure 8:**
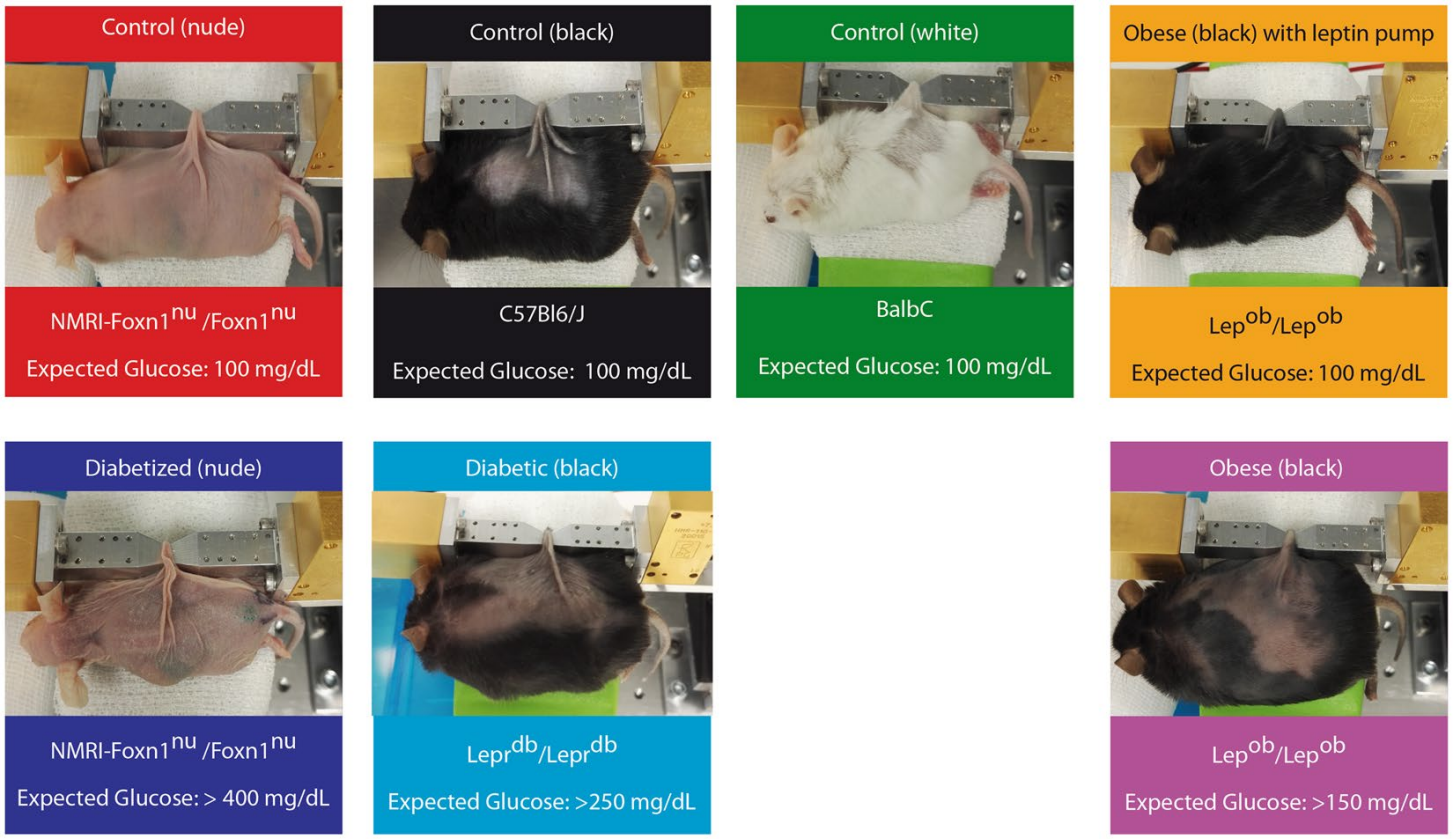
List of analyzed animals.

A list of the mice strains employed in the experiments can be found in Fig. 8. The mice were purchased from Elevage-Janvier (France) and housed individually in pathogen-free conditions at the Centro de Investigaciones Energéticas, Medioambientales, y Tecnológicas (CIEMAT) Laboratory Animals Facility (Spanish registration number 28079–21A). Beside the use of genetic healthy, diabetic and obese mice strains, three of the nude control mice were diabetized by injecting Streptozotocin (Sigma-Aldrich, Inc., Missouri, USA) during three consecutive days and two of the obese animals were treated with a 28-day lasting micro osmotic pump containing human leptin. The diabetized mice were 12 days under the diabetized condition and the leptin-treated mice were 25 days under this condition before the experiments were performed.

### Legal Requirements

All experimental procedures were carried out according to European and Spanish laws and regulations (European convention ETS 1 2 3, about the use and protection of vertebrate mammals used in experimentation and other scientific purposes, Directive 2010/63/UE and Spanish Law 6/2013, and R.D. 53/2013 about the protection and use of animals in scientific research). Procedures were approved by the Animal Experimentation Ethical Committee of the CIEMAT according to all external and internal bio-safety and bio-ethics guidelines, and by Spanish competent authority with registered number PROEX 176/15.

### Mm-wave measurement and calculation of the complex scattering parameters

To perform a reflection and transmission measurement the mice were anesthetized with a standard rodent anesthesia (ketamine/medetomidine) to minimize psychological stress for the animals and to avoid unexpected movements of the animals. A skin fold was placed between two straight cuts of a WR10 waveguide. It is important that the distance of the two waveguides was constant for every animal to avoid differences in the transmission phase due to different lengths. The acquisition of the amplitude and phase of the reflection and the transmission lasted around 45 seconds across the full W-band between 75 GHz and 110 GHz, which employed a frequency multiplier to multiply the initial signal frequency from 12.5 GHz to 18.5 GHz into the W-band. The wave propagates through a dual-directional coupler, which has been directly connected to the waveguide structure close to the skin of the mice. No antenna was used to eliminate parasitic reflection effects. The transmission through the skin, the reflection back into the bidirectional coupler and the reference wave, which is important for the calculation of the scattering parameters, were detected with three HMR-110-6 W-band receivers (Radiometer Physics GmbH, Meckenheim, Germany). The frequency multiplier AFM6-110, the dual-directional coupler and the three receivers all stem from Radiometer Physics GmbH, Meckenheim, Germany. For the generation of the K_u_ band frequencies two ABSYN420 synthesizers (AnaPico, Zurich, Switzerland) were used in steps of 250 MHz. Between the generation frequency of the frequency mulitplier and the local oscillator frequency of the receivers an offset of 1.5 MHz was used, to produce a multiplication factor of ×6, resulting in an intermediate frequency of 9 MHz. The data analysis was performed with a Labview program working as a multi-channel lock-in amplifier to record the amplitude of the three signal channels and the phase differences of the data channels transmission and reflection according to the reference channel.

For the calculation of the scattering parameters a basic calibration was performed. To simulate a transmission thru standard the two rectangular waveguides were brought into contact to have the best possible transmission (Thru). Special care was taken to align both parts to each other in order to avoid any potential reflections at the interface between the two arms. For the calibration of the reflection channel a polished metal plate was jammed between the waveguide cuts for the highest possible reflection (Short).

The rudimentary Thru standard should produce no significant phase shift and the highest possible transmission amplitude, the Short standard should provide a phase shift of 180° and maximum reflection amplitude. The measurements of the skin fold were corrected with these two standards to create the scattering parameters of the system. More elaborate calibration procedures are possible, like the one using offset short calibration procedures or Thru-Reflect-Line (TRL) calibration methods. The calibration was repeated before performing measurements on each mouse.

1 $${A}_{S11}[dB]=20\cdot \mathrm{log}(\frac{{A}_{{\rm{Re}}fl,corr;Mouse}}{{A}_{{\rm{Re}}fl,corr;Short}})$$

2 $${\phi }_{S11}[^\circ ]=180^\circ +{\phi }_{{\rm{Re}}fl,corr;Mouse}[^\circ ]-{\phi }_{{\rm{Re}}fl,corr;Short}[^\circ ]$$

3 $${A}_{S21}[dB]=20\cdot \mathrm{log}(\frac{{A}_{Trans,corr;Mouse}}{{A}_{Trans,corr;Thru}})$$

4 $${\phi }_{S21}[^\circ ]={\phi }_{Trans,corr;Mouse}[^\circ ]-{\phi }_{Trans,corr;Thru}[^\circ ]$$

### Data availability

The datasets generated and analysed during the current study are available from the corresponding author on reasonable request.

## References

[1] Kruyt ND, Biessels GJ, Devries JH, Roos YB (2010). Hyperglycemia in acute ischemic stroke: pathophysiology and clinical management. Nat. Rev. Neurol..

[2] Milicevic Z (2008). Natural history of cardiovascular disease in patients with diabetes: role of hyperglycemia. Diabetes Care.

[3] Brownlee M (2005). The pathobiology of diabetic complications: a unifying mechanism. Diabetes.

[4] American Diabetes Association, Diagnosis and classification of diabetes mellitus. *Diabetes Care* **32**, 62–67 (2009).

[5] Kuo I-C (2016). Glycated Hemoglobin and Outcomes in Patients with Advanced Diabetic Chronic Kidney Disease. Sci. Rep..

[6] Fraser DA, Hansen KF (2005). Making sense of advanced glycation end products and their relevance to diabetic complications. Inter Diabetes Monit..

[7] Gkogkolou P, Böhm M (2012). Advanced glycation end products: Key players in skin aging?. Dermatoendocrinol.

[8] Brownlee M, Vlassara H, Cerami A (1984). Nonenzymatic glycosylation and the pathogenesis of diabetic complications. Ann. Intern. Med..

[9] Singh VP, Bali A, Singh N, Jaggi AS (2014). Advanced glycation end products and diabetic complications. Korean J. Physiol. Pharmacol..

[10] Murillo J (2008). Advanced glycation of type I collagen and fibronectin modifies periodontal cell behavior. J. Periodontol..

[11] Duran-Jimenez B (2009). Advanced glycation end products in extracellular matrix proteins contribute to the failure of sensory nerve regeneration in diabetes. Diabetes.

[12] Lee S-Y (2013). Glycosylated hemoglobin and albumin-corrected fructosamine are good indicators for glycemic control in peritoneal dialysis patients. PLoS One.

[13] Furusyo N, Hayashi J (2013). Glycated albumin and diabetes mellitus. Biochim. Biophys. Acta.

[14] Roohk HV, Zaidi AR (2008). A review of glycated albumin as an intermediate glycation index for controlling diabetes. J. Diabetes Sci. Technol..

[15] Yang C (2012). Glycated albumin is a potential diagnostic tool for diabetes mellitus. Clin. Med..

[16] Hernandez-Cardoso G (2017). Terahertz imaging for early screening of diabetic foot syndrome: A proof of concept. Sci. Rep..

[17] Martín-Mateos P (2016). *In-vivo*, non-invasive detection of hyperglycemic states in animal models using mm-wave spectroscopy. Sci. Rep..

[18] List EO (2007). *u*. *a*. Analysis of mouse skin reveals proteins that are altered in a diet-induced diabetic state: A new method for detection of type 2 diabetes. Proteomics.

[19] Algenstaedt P (2003). *u*. *a*. Microvascular alterations in diabetic mice correlate with level of hypeglycemia. Diabetes.

[20] Ibuki A (2012). *u*. *a*. Skin fragility in obese diabetic mice: Possible involvement of elevated oxidative stress and upregulation of matrix metalloproteinases. Exp. Dermatol..

[21] Yosipovitch G, DeVore A, Dawn A (2007). Obesity and the skin: Skin physiology and skin manifestations of obesity. J. Am. Acad. Dermatol..

[22] Pasparakis M, Haase I, Nestle FO (2014). Mechanisms regulating skin immunity and inflammation. Nat. Rev. Immunol..

